# Reading Pictures for Story Comprehension Requires Mental Imagery Skills

**DOI:** 10.3389/fpsyg.2016.01630

**Published:** 2016-10-24

**Authors:** Inouk E. Boerma, Suzanne E. Mol, Jelle Jolles

**Affiliations:** ^1^Department of Educational Neuroscience, Faculty of Behavioural and Movement Sciences, Vrije Universiteit AmsterdamAmsterdam, Netherlands; ^2^Department of Language Didactics, iPabo University of Applied SciencesAmsterdam, Netherlands; ^3^Clinical Child and Adolescent Studies, Faculty of Social and Behavioural Sciences, Leiden UniversityLeiden, Netherlands

**Keywords:** mental imagery, reading comprehension, mental model, narrative, pictures, primary school, multimedia effect

## Abstract

We examined the role of mental imagery skills on story comprehension in 150 fifth graders (10- to 12-year-olds), when reading a narrative book chapter with alternating words and pictures (i.e., text blocks were alternated by one- or two-page picture spreads). A parallel group design was used, in which we compared our experimental book version, in which pictures were used to replace parts of the corresponding text, to two control versions, i.e., a text-only version and a version with the full story text and all pictures. Analyses showed an interaction between mental imagery and book version: children with higher mental imagery skills outperformed children with lower mental imagery skills on story comprehension after reading the experimental narrative. This was not the case for both control conditions. This suggests that children’s mental imagery skills significantly contributed to the mental representation of the story that they created, by successfully integrating information from both words and pictures. The results emphasize the importance of mental imagery skills for explaining individual variability in reading development. Implications for educational practice are that we should find effective ways to instruct children how to “read” pictures and how to develop and use their mental imagery skills. This will probably contribute to their mental models and therefore their story comprehension.

## Introduction

Understanding a story text requires the reader to form a mental representation, or situation model, of that story (e.g., Kintsch, 1988; Zwaan and Radvansky, 1998). Mental imagery, i.e., the ability to create mental images of the story in your “mind’s eye,” can enhance the quality of these mental models (Algozzine and Douville, 2004; Mar, 2004; De Koning and Van der Schoot, 2013), and therefore contributes to successful reading comprehension. Pictures can facilitate the creation of a mental representation as well, as they can clarify implicit or unclear relations in the text (e.g., Eitel and Scheiter, 2015). In addition, it is claimed that adding pictures to a text results in deeper learning (Mayer, 2002b; Schnotz, 2002; Schnotz and Bannert, 2003; Schüler et al., 2015). This so-called “multimedia effect” (e.g., Mayer, 2002b) has especially been found in research with expository texts (e.g., Levie and Lentz, 1982; Peeck, 1993; Carney and Levin, 2002; Mayer, 2002a), but there has been little evidence in the case of narrative texts (e.g., Newton, 1995; Hannus and Hyönä, 1999; Slough et al., 2010). Narrative texts form an important genre though, as they act as a model of the human social world and allow readers to experience thoughts and emotions that are evoked by the story events (Mar and Oatley, 2008). In this quasi-experimental study, we examined the role of elementary school children’s mental imagery skills in their ability to understand a story that required an integration of words and pictures. More specifically, we examined a special kind of narrative in which pictures took a different position than in standard illustrated children’s books, by providing additional and unique information that was not expressed in words. We expected that understanding this experimental narrative would put high demands on both children’s mental imagery skills, and their ability to “read” pictures.

When readers engage in a story, they create and update their mental model while reading (Johnson-Laird, 1983; Kintsch, 1988; Glenberg and Langston, 1992). Mental models are thought to include the information that is explicitly mentioned in the text (“textbase”) and the situational representation of story events (e.g., information about the people, locations, and actions described in the story). This information is combined with the reader’s knowledge about stories in general (e.g., structure, perspective) as well as with the reader’s background knowledge about the topic of the story (Kintsch, 1988; Schnotz, 2002; Zwaan and Singer, 2003). While readers engage with a story, they create mental visual (and other sensory) images, or “internal pictures” of the story, which facilitates mental model making (Johnson-Laird, 1998; Schnotz, 2002; Schnotz and Bannert, 2003; Algozzine and Douville, 2004). In addition, readers use mental imagery to infer what is in the characters’ mind, to predict and explain their actions and emotions (i.e., mentalizing), which adds to the mental model as well (e.g., Gernsbacher et al., 1992; Mar et al., 2006; Mar, 2011).

Research among students of various ages has shown that mental imagery skills play a role in explaining individual differences in reading comprehension. Students who are not inclined to make mental images in general while reading were found to show poor reading performance, whereas students who are used to making mental images tended to be more proficient readers (Bell, 1991; Snow, 2002; Hibbing and Rankin-Erickson, 2003; De Koning and Van der Schoot, 2013). It has been suggested that mental imagery is an effective strategy for remembering and connecting story elements, and for concretizing complex and abstract ideas from the text (Bell, 1991; Gambrell and Jawitz, 1993; Algozzine and Douville, 2004; De Koning and Van der Schoot, 2013). The exact way in which visual images are created in the brain is not yet clear (see for example, Kosslyn, 1994; Kosslyn et al., 2001, for an interesting discussion on this topic). Importantly, mental imagery seems to be particularly invoked by narrative texts. As narratives can be seen as a simulation of the social world, readers are more likely to feel connected to story events and characters (Mar and Oatley, 2008). This engagement with the story is thought to enhance the quality of readers’ mental models as well as their story comprehension (Green et al., 2004).

It has repeatedly been shown that readers learn and understand more from a text when pictures are added, which has been referred to as the “multimedia effect” (Mayer, 2002b; Schnotz, 2002; Schnotz and Bannert, 2003; Schüler et al., 2015). This can be explained in two ways. First, pictures can aid mental model making, in a way comparable to mental imagery. While words can be seen as “descriptive” representations, composed of symbols with an arbitrary structure, pictures, in contrast, are “depictive” external representations, showing the meaning or the content that they represent (Schnotz, 2002; Bartholomé and Bromme, 2009). As such, pictures provide an initial framework, on which readers can base their mental model (Eitel and Scheiter, 2015). Adding pictures to a text might be especially helpful for poor learners, because pictures can make relations in the text explicit and resolve ambiguous information in the text (Levie and Lentz, 1982; Schnotz and Bannert, 2003; Eitel and Scheiter, 2015). Moreover, pictures may serve as clues for readers to decide which parts of the text are important to keep activated in the mental model (Glenberg and Langston, 1992; Pike et al., 2010). This will foster readers’ comprehension of a text (Pike et al., 2010).

Second, it is claimed that the contribution of both verbal and pictorial information results in a deeper processing of the text, which is thought to benefit reading comprehension (Bartholomé and Bromme, 2009; Schüler et al., 2015). A text that contains pictures provides learners with two different sources of information, which has been referred to as “dual coding” (Paivio, 2006). The processing of words and pictures, which can be seen as equally complex (Arizpe and Styles, 2003; Walsh, 2003), leads to multiple representations in the brain. Combining these representations is thought to lead to deeper learning and increased comprehension. There is no consensus yet on the way the information from text and pictures is combined. According to some researchers, the verbal and pictorial information remain two separate, though interconnected subsystems, an assumption that is grounded in Paivio’s dual coding theory (Mayer, 1997, 2002a,b; Paivio, 2006). Others claim that the two types of representations are merged into a single representation. This results in a richer mental representation of the story, and therefore enhanced comprehension (Schnotz and Bannert, 2003; Schüler et al., 2015).

While both mental imagery and pictures have been shown to contribute to reading comprehension, not many studies have examined the interaction between mental imagery skills and “picture reading ability” in predicting reading comprehension. It is therefore not quite clear how various levels of mental imagery skills contribute to the comprehension of texts with pictures. On the one hand, it is thought that especially readers with low mental imagery skills may benefit from pictures accompanying a text, because these pictures can help them form a mental model of the story (Glenberg and Langston, 1992; Hibbing and Rankin-Erickson, 2003). On the other hand, readers with higher mental imagery skills might be better able to integrate the information from two different sources into one mental model (Bell, 1991; Peeck, 1993; Paivio, 2006). That would suggest that adding pictures to a text would be especially beneficial in the case of readers with high imagery skills. This is in line with the idea that for constructing both external and internal pictures (i.e., mental images), the same underlying processes of selecting, combining, and organizing information are involved (Leopold and Mayer, 2015). The latter view is supported by a study that did examine the interaction between mental imagery skills and picture reading ability in texts (Gambrell and Jawitz, 1993). Readers in this study who used mental imagery skills and pictures in interconnected ways, showed enhanced text comprehension. These findings suggest a positive interaction between mental images and pictures. In the current study, this interaction is further explored.

So far, research has been focused mainly on texts in which pictures were added as some kind of supplementary material. In this quasi-experimental study, we are interested in what will happen to children’s comprehension when they are confronted with a different kind of text, i.e., a text in which pictures occasionally replace words. We created a unique experimental storybook for 10- to 12-year-old children, in which parts of the story were told only in the form of illustrations. In contrast to the pictures in graphic novels and comic books, the illustrations in our experimental storybook did not contain any words, i.e., there were no captions. In order to understand such a text, children need to combine the information they derived from the pictures with information they derived from the written text into their mental model. We compared children’s story comprehension of our experimental narrative to two control conditions: (1) a version of the narrative that contains the complete text of the story plus all additional pictures; (2) a version of the narrative, consisting of the full text only, without any pictures.

The current study set out to investigate the role of children’s mental imagery skills, when reading a text in one of these three test conditions. Previous studies have suggested relations between the processing of external and internal images (Gambrell and Jawitz, 1993; Leopold and Mayer, 2015). We therefore expected that children’s mental imagery skills are related to their ability to “read” pictures and to combine two sources of information (pictures and text) into a complete understanding of a story. We hypothesized that children’s comprehension of the experimental narrative is related to individual differences in mental imagery skills. In other words, children who self-report that they are, in general, not used to creating (many) mental images when they are reading were expected to experience more difficulties with the experimental narrative, compared to children with higher mental imagery skills. Contrasting children’s comprehension of our experimental narrative to the two control conditions allows us to take a closer look at the difficulty that children with different levels of mental imagery skills may experience when integrating text and pictures. We chose fifth graders as our participants, because we were interested in examining readers who had passed the early stages of reading development.

## Materials and Methods

### Participants

The participants in this study were 150 fifth graders (72 boys, 78 girls) from ten different primary schools. One class per school participated, and between 6 and 22 students per class participated in the study. The schools were situated in the north-western part of the Netherlands in the greater-Amsterdam region and were part of the same school district. They can be considered mainstream schools. Students were between 10 and 12 years old (*M*_age_ = 11.1 years, *SD* = 0.39). Children with learning or behavioral problems (e.g., dyslexia, AD(H)D), as reported by their teachers, were excluded from the sample (*n* = 21), which reduced the original sample of 171 children to 150 children.

### Reading Materials

For this study, we used the first chapter of a Dutch children’s book that had not yet been published at the time of the data collection. The book was written by a respected Dutch children’s book author, in collaboration with a well-known illustrator. The book contained a fictitious narrative story about a boy growing up with his father, a samurai, in Japan (Kouwenberg, 2014). Three different versions of the same narrative were created: the experimental picture version; the pictures-and-text version; and the text-only version. To illustrate the differences between these three versions we have included sample pages in Appendices A to C. These pages were taken from near the end of chapter 1. Likewise, the corresponding story comprehension questions are given in Appendix D.

#### Experimental Picture Version

In the experimental narrative, the story (18 pages) was partly told in words (about 1,800), and partly in pictures (nine full pages). Text blocks (between about 150 and 750 words) were alternated by one- or two-page picture spreads. These pictures replaced textual information of about 200 words. For example, in the sample pages of the experimental picture version (see Appendix A), the paragraphs describing Kodo’s final successful training were deleted and replaced by a two-page picture spread. Children were required to infer the important information from that picture. Sometimes, important “missing” textual information that could not be captured in the picture is summarized in a few sentences at the beginning of the next paragraph. Despite these little summaries, the experimental picture version required the children to look at the pictures very carefully in order to get a full comprehension of the text, since the story was continued in the pictures. This version was pilot-tested with individual children by the author of the book at a Dutch primary school and the children’s reactions were filmed and analyzed (Kouwenberg, 2013). The pilot research showed that children were able to understand this interplay between text and pictures and that, in general, they enjoyed reading the story.

#### Pictures-and-Text Version

The pictures-and-text narrative (23 pages) consisted of the same pictures that were used in the experimental narrative plus the whole text of the story (about 2,500 words). The text was alternated by corresponding one- or two-page picture spreads (see Appendix B).

#### Text-Only Version

The text-only narrative (14 pages) consisted of the complete text of the story (about 2,500 words) and no pictures (see Appendix C).

### Procedure

A parallel group design was employed to compare the three versions of the narrative. Children with parental consent from one classroom all received the same book version to prevent them from being distracted by the noticeable differences among book versions. Classrooms were randomly assigned to one of the versions. The distribution of boys and girls was relatively equal across book versions. Two group test sessions were organized, led by the researcher and a research assistant. During the first session, the children received a self-report questionnaire, which included scales that tapped into children’s imagery skills and social-economic status (SES). This session lasted 15–30 min. In addition, schools provided us with the standard test scores (standard reading test scores and oral reading fluency test scores) of the participating children who had parental consent.

During the second session, about 1 week later, children participated in the experiment. The experimental picture version was read by 47 children (31.3% in total; 46.8% boys, 53.2% girls; three classrooms); the pictures-and-text version by 54 children (36.0% in total; 46.3% boys, 53.7% girls; four classrooms); and the text-only version by 49 children (32.7%; 51.0% boys, 49.0% girls; three classrooms). Children were instructed to carefully read the story, and to take as much time as they wanted, because they were supposed to answer the comprehension questions without having access to the text. Additional instructions were given in classrooms in which the experimental picture version was read. Children in these classrooms were told that they were about to read a different kind of book. They were instructed to carefully read the words and look at the pictures, because both contributed to the story.

Directly after handing in the booklet, children received another booklet with 28 multiple-choice questions about the story. During this session, which had a maximum duration of 60 min, all children were seated at individual desks to prevent them from copying each other’s work and from talking to each other.

According to the Ethical Principles of our university, a study on a group of people, such as students in a classroom is exempt from individual informed consent. Such consent was, however, given by parents, children, teachers, and the school board. In addition, children were informed that they could stop participating at any time they wished.

### Questionnaire

A self-report questionnaire was developed to evaluate children’s mental imagery skills and SES. This questionnaire was administered to the children during the first session.

#### Mental Imagery

Children answered nine questions about mental imagery, that tapped into both the visual aspect (e.g., *When I read, it’s like as if I see the people in the story*), and the mentalizing aspect (e.g., *When someone in the story is happy, I can almost feel that myself*). These items were developed by Tellegen and Frankhuisen (2002), and were validated in a large sample of Dutch fourth, sixth, eighth, and tenth grade students. The questionnaire aimed to measure children’s general self-reported ability to create mental images while reading. The students were instructed to choose the answer that best described themselves, ranging from never (=1) to always (=4). A mean score was created for each child (*M* = 2.34, *SD* = 0.69, range = 1.11–4.00) and a higher score on this measure reflects more self-reported imagery skills. One missing value per participant was allowed when creating the mean score (2% had one missing value). The scale showed a good internal consistency: Cronbach’s α = 0.89.

#### Social-Economic Status (SES)

To establish Social-Economic Status, the four questions of the Family Aﬄuence Scale (Boyce et al., 2006) were used, that was especially created for children. For each participant a composite SES score was created following the authors’ instructions. In our sample, a total mean score of *M* = 6.75 was obtained (*SD* = 1.86), with scores ranging between 3 and 9. According to Boyce et al. (2006), these scores can be translated into 0% children with a low SES, 22.0% with a medium SES, and 76.7% with a high SES (we had missing values for two children).

### Reading Ability Measures

#### Standard Reading Test

We used standard reading test scores of reading comprehension tests (Cito-tests; Weekers et al., 2011) that are administered in February at almost every school in the Netherlands, including all participating schools. The test scores are an important source of information for teachers to decide how to differentiate their reading instruction. Raw scores on these tests are converted by a computer program to ability scores and norm-referenced scores, which allows comparison between schools. In our study, we used children’s ability scores (*M* = 48.71, *SD* = 15.61, range = 17–122). The standard reading test consists of a booklet with a number of texts that children have to read and answer comprehension questions about.

#### Oral Reading Fluency

As an indication for children’s oral reading fluency, we used another Dutch standard test that is administered twice a year (in February and June) at almost every school in the Netherlands, including all participating schools. Children are asked to read as many (isolated) real words as they are capable of within 1 min (DMT-tests; Krom et al., 2010). Test scores are based on fluency and accuracy and provide schools with ability and norm-referenced scores, comparable to the standard reading test mentioned above. The scores we used in our study were acquired in June, which was around the time of the experiment. The mean score for our participants was 99.52 (*SD* = 13.29, range = 61–133).

#### Story Comprehension Test

We carefully developed a story comprehension test with 28 multiple-choice questions with four answer possibilities about the story the children read during the experimental test session in June. One question in the task was deleted, because it appeared that this question was easier to answer for children who read the experimental picture version (which explicitly stated the answer in the text), compared to children who read one of the two control versions (who had to make an inference in order to be able to answer that specific question). Therefore, the story comprehension score is based on the 27 remaining questions. Some questions were on information that was explicitly mentioned in the text (e.g., example question 3 and 4 in Appendix D), and other questions required the children to make inferences which are an indication of an overall understanding of the story (e.g., example question 1 in Appendix D). The comprehension test was pilot tested with fifth graders with different levels of reading ability, and this resulted in some changes in wording.

Children had to complete the test without having the text at their disposal, so they were not able to look up the answer. One child was excluded from the dataset, because he skipped two pages (containing 12 questions) of the test. Total story comprehension scores varied between 9 and 27 correct answers (*M* = 20.11, *SD* = 3.82). Reliability analyses showed a Cronbach’s alpha of α = 0.72. The Pearson’s correlation coefficient between this story comprehension score and the standard reading test was significant and positive (*r* = 0.47, *p* < 0.001), implying that children who were good at reading comprehension in general also scored higher on our story comprehension measure.

### Statistical Analyses

To analyze the data, we used the statistical package SPSS (version 23). We first performed correlation analyses and independent samples *t*-tests in order to determine which variables should be included as covariates in subsequent analyses. Since we randomly assigned classes to a test condition, and students within classes share (teaching) experiences that might influence their reading performance, we decided to perform multilevel analyses. The intraclass correlation showed that class explained only 2.8% (ρ = 0.03) of the variance in children’s story comprehension. However, since our design was hierarchically structured, we decided to use multilevel analyses, nesting students within classrooms. We used restricted maximum likelihood estimation, because we were not interested in comparing several models. As fixed effects, we included our covariates, mental imagery, test condition, and the interaction between mental imagery and test condition. Classroom was added as our random intercept.

## Results

First, correlations were calculated between the continuous variables (**Table 1**). Since we did not find any significant correlations for SES, we did not control for this measure in our analyses. We did control for sex, because independent samples *t*-tests (**Table 2**) showed sex differences in mental imagery [*t*(148) = -2.04, *p* = 0.044] and standard reading scores [*t*(146) = -3.76, *p* < 0.001]. For both variables girls significantly outperformed boys. We also checked for any pre-existing differences between children who were randomly assigned on classroom level to our three test conditions and we found differences in oral reading fluency [*F*(2,147) = 8.28, *p* < 0.001]. Therefore, we included sex and oral reading fluency as covariates in all our analyses.

**Table 1:** Correlations between SES and reading scores.

	1	2	3	4	5
1. SES	X				
2. Standard reading test	0.06	X			
3. Oral reading fluency	0.05	0.29^∗∗^	X		
4. Story comprehension	0.10	0.46^∗∗^	0.31^∗∗^	X	
5. Mental imagery skills	0.09	0.34^∗∗^	0.32^∗∗^	0.26^∗∗^	X

*^∗^*p* < 0.05, ^∗∗^*p* ≤ 0.01, ^∗∗∗^*p* < 0.001.*

**Table 2:** Sex differences on SES and reading scores.

	Boys		Girls	
	*M*	*SD*	*M*	*SD*
SES	6.76	1.56	6.74	1.62
Standard reading test^∗∗∗^	43.90	12.80	53.14	16.69
Oral reading fluency	98.60	12.45	100.37	14.04
Story comprehension	19.83	3.54	20.36	4.06
Mental imagery skills^∗^	2.22	0.65	2.45	0.71

*^∗^*p* < 0.05, ^∗∗^*p* < 0.01, ^∗∗∗^*p* < 0.001.*

### Imagery and Pictures Replacing Text in Relation to Story Comprehension

**FIGURE 1:**
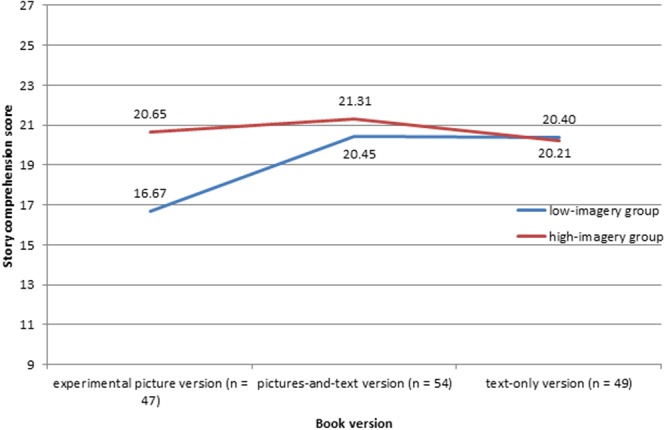
**Interaction between imagery skills and test condition, controlling for oral reading fluency and gender**.

Our multilevel analyses (**Table 3**) showed that the interaction between imagery skills and test condition significantly predicted children’s story comprehension [*F*(2,140.05) = 3.38, *p* = 0.037]. This indicates that the effect of children’s imagery skills on their story comprehension scores depended on the book version they read. The parameter estimates showed that the imagery skills of children who read the experimental picture version significantly contributed to their story comprehension [*b* = 2.54, *SE* = 1.15, *t*(137.51) = 2.20, *p* = 0.029], compared to children who read the text-only version. There were no differences between children who read the pictures-and-text version and children who read the text-only version [*b* = 0.47, *SE* = 1.18, *t*(140.57) = 0.40, *p* = 0.688]. In **Figure 1**, the interaction is visualized, based on a median split on children’s mental imagery skills (median = 2.22).

**Table 3:** Fixed effects estimates predicting children’s story comprehension (*N* = 150 individuals from 10 schools).

	*Estimates*	*SE*	*95% CI*
**Fixed effects**			
Intercept	14.01	2.87	8.33, 19.69
Sex (=boys)	-0.09	0.59	-1.25, 1.07
Oral reading fluency	0.07^∗∗^	0.02	0.02, 0.11
Mental imagery skills	0.04	0.92	-1.79, 1.86
Test condition (=1)	-7.85^∗∗^	2.66	-13.12, -2.58
Test condition (=2)	-1.33	2.76	-6.79, 4.13
Interaction test condition (=1) ^∗^ mental imagery skills	2.54^∗^	1.15	0.26, 4.82
Interaction test condition (=2) ^∗^ mental imagery skills	0.47	1.18	-1.85, 2.80
**Random effects**			
Residual	11.78^∗∗∗^	1.43	9.29, 14.94
Intercept	0.34	0.58	0.01, 9.72
Estimated parameters		10	
-2 restricted log likelihood		789.97	

*Test condition 1 = experimental picture version; test condition 2 = pictures-and-text version; test condition 3 = text-only version.*

*^∗^*p* < 0.05, ^∗∗^*p* < 0.01, ^∗∗∗^*p* < 0.001.*

The same pattern was found when we computed the correlation between children’s mental imagery and story comprehension for the three conditions separately. There only was a moderate relation for the experimental picture version (*r* = 0.55, *p* < 0.001 for bivariate correlations; *r* = 0.53, *p* < 0.001 for partial correlations, controlling for gender and oral reading fluency), whereas no significant relations between mental imagery skills and story comprehension were found for the text-only and the pictures-and-text condition. This shows that children with higher imagery skills were better able to understand the story in the experimental picture condition, whereas imagery skills did not affect their comprehension of the story in the control versions.

## Discussion

The aim of the present study was to examine the role of mental imagery skills when reading a narrative text with alternating pictures and words. To do so, we examined fifth graders’ comprehension of an experimental book version in which the words were partly replaced by pictures and compared this to two control groups, who read a book version with only text or with all text and pictures. We found an interaction between book version and mental imagery skills. Children with lower imagery skills had problems understanding our experimental book version, whereas this was not the case for our control conditions. This implies that children who are more experienced in using mental imagery during reading were able to build a mental model that helped them connect pictures and words in such a way that this enhanced their understanding of this experimental narrative. Children with lower imagery skills, in contrast, were less skilled at this procedure and may not have been able to integrate words and pictures into a meaningful story. Consequently, they experienced comprehension difficulties.

Our results particularly show that reading a narrative that requires a correct interpretation of the pictures, and a correct integration of these pictures with the text places high demands on imaginative abilities (Bell, 1991; Peeck, 1993; Schnotz, 2002; Paivio, 2006). In the experimental version, children were required to look at the pictures carefully and integrate the core information from those pictures with the text in order to get full understanding of the story. For example, the sample pages in Appendix A show a scene, in which Kodo is practicing his fighting skills with his father. Children should combine the words describing the events that happened before (the first fighting practices) with the displayed picture and the words that follow the picture. From this they should infer that the practice displayed in the picture went better. For children who have trouble forming images or making a movie in their “mind’s eye” while reading, this might be a task that is too difficult to perform successfully. This will, however, lead to an incomplete understanding of the story and to lower performance on the story comprehension task. Imagery skills might be especially important in the comprehension of texts in which a lot of the information is not explicitly stated, since those texts require more work from the reader. Overall, children with lower imagery skills (using a median split) who read the experimental version achieved a score of 62% correct, whereas the low-imagery group who read one of the control conditions had a score of 76% correct. An example of a question that reflects this pattern is example question 2 (see Appendix D). Children with lower imagery skills who read the experimental version found this question harder (*n* = 21; 67% answered correctly) than children who read one of the control conditions (*n* = 57; 77% answered correctly). Interestingly, this question is not directly connected to a specific part of the text that has been replaced by a picture in the experimental version. We assume that this is because many questions in the story comprehension test require an overall understanding of the text. Future studies might examine the questions in our story comprehension test in more detail, for example with the items of the test included as a level in a multilevel analysis. This might show different patterns of questions answered correctly by children with higher versus lower imagery skills when reading the experimental narrative. Finding such patterns would shed more light on the way mental imagery skills aid in understanding a story that requires an integration of information from text and pictures.

It has been reported repeatedly that individual differences in mental imagery skills are related to differences in reading comprehension (Bell, 1991; Snow, 2002; Hibbing and Rankin-Erickson, 2003; De Koning and Van der Schoot, 2013). However, in our study we found that this was only the case for the experimental picture version. Contrary to our experimental narrative, our two control versions stated all information in words. Such “concrete” stories are thought to require less reading between the lines and to elicit mental imagery more easily (Carney and Levin, 2002; Paivio, 2006). For the pictures-and-text and the text-only versions, performance on the story comprehension task was not affected by mental imagery skills. Perhaps the information in these two book versions was so concrete that individual differences in mental imagery skills did not contribute to a differential story comprehension task performance. However, we did not examine our narratives on explicitness of information. Future research might examine texts that differ in the explicitness of information that is provided.

In this study, we used the first chapter of an original fictive narrative children’s story, which is a relatively long text, compared to the short stories or sentences that are often used in reading research. Therefore, we assume that our results provide a realistic view on the way children use their mental imagery skills to interpret “authentic” stories composed of text and pictures. Interestingly, our study did not show an advantage for children who read the story with full text and pictures, for we found no difference in story comprehension scores between children who read the pictures-and-text version and the text-only version. According to the multimedia effect, children who read the pictures-and-text version should have had an advantage as processing both pictures and text should result in deeper learning (Mayer, 2002b; Schnotz, 2002; Schnotz and Bannert, 2003; Schüler et al., 2015), but that was not the case. It may be that children who read the pictures-and-text version did not actively process the pictures to understand the storyline, because the story in words was already clear and engaging enough for them (Carney and Levin, 2002). Another reason might be that the condition of spatial contiguity was not met. According to the so-called “spatial contiguity principle,” text and pictures should be presented close to each other for a positive effect on learning (Mayer, 1997, 2002b). In our materials, however, text was alternated with one- or two-page picture spreads. Importantly, the order of presentation (i.e., text before picture or picture before text) does not seem to affect comprehension (Eitel and Scheiter, 2015).

Our study has some important implications for educational practice, focusing both at children’s “picture reading” and mental imagery skills. First, since our results have not shown the added value of pictures accompanying a story, it might be useful to provide trainings to teach children how to “read” the pictures they encounter in order to improve their reading comprehension. Such visual literacy trainings are not common in educational practice, however (De Koning and Van der Schoot, 2013; Ploetzner et al., 2013), especially when it comes to the reading of narratives. When picture instructions are given, they tend to be non-specific, not indicating what one should be looking at in the illustration. That is, it is unlikely that children will capture the full meaning of a (complex) illustration, when they are merely told to pay attention to it (Levie and Lentz, 1982; Weidenmann, 1989; Peeck, 1993; Ploetzner et al., 2013). While the global meaning of a picture can usually be understood in a rapid way, full and deep understanding of the picture, on a higher order level, requires more cognitive effort (Peeck, 1993; Avgerinou and Ericson, 1997; Fei-Fei et al., 2007; Ainsworth, 2008; Bartholomé and Bromme, 2009; Ploetzner et al., 2013). Furthermore, such trainings should include a focus on metacognitive skills. Children should be taught to monitor their understanding of the story, including the illustrations (Peeck, 1993). Since learners tend to perceive the processing of pictures as relatively easy, they will probably not be able to monitor their understanding properly (Ainsworth, 2008). This is reflected in the overall lower reading comprehension score of children who read our experimental picture version. These children might not have taken enough effort to fully comprehend the pictures they encountered while reading the story.

A second implication follows from our findings on the importance of mental imagery skills. Teaching children how to use mental imagery will improve their imagery skills (Hibbing and Rankin-Erickson, 2003; Algozzine and Douville, 2004; De Koning and Van der Schoot, 2013), which can contribute to better reading performance as well. Particularly children with poor imagery skills can benefit from these trainings (Avgerinou and Ericson, 1997). In order for a mental imagery training to be successful, teachers need to model mental imagery by thinking-out-loud and providing children with feedback on their imagery (Van de Ven, 2009). Previous research has indeed found that a combination of mental imagery strategy training and visual literacy training was the most successful approach for improving reading comprehension of fourth-graders (Gambrell and Jawitz, 1993; Woolley, 2007). Experimental research on finding effective ways to teach children how to successfully deal with visual information and to develop mental imagery skills is limited though.

There are some issues that could be taken into account when interpreting our findings. First, children in our study had no access to the story text while answering the story comprehension questions, because we were explicitly interested in their ability to form a mental representation of the text. We expect that the absence of the text did not lower their performance, since we have informed children in advance about the comprehension test and previous research has shown that children’s reading performance is not affected when children are informed in advance (Schaffner and Schiefele, 2013). Second, a self-report scale was used to measure mental imagery skills. Research with young adults has shown that such self-report scales measure the same underlying construct as fMRI-tasks of mental imagery (Cui et al., 2007). However, future work is required to replicate this finding with younger children. Third, it might be that our relatively long narrative evoked much mental imagery (Mar and Oatley, 2008), so that the children did not look at the pictures carefully, because these interfered with their own mental images (Schnotz and Bannert, 2003). We did not ask children about the mental images they created while they were reading the book chapter and whether or not their images matched with the pictures in the book. It would be interesting to replicate this study by also including a behavioral measure of mental imagery, and assessing children’s accuracy and reaction time in creating mental images. Future studies might also include a measure of working memory, as it has been shown that working memory is involved in understanding visual narratives (see, for example, Magliano et al., 2015). A final issue concerns the type of text examined. Children are not very likely to come across narratives like our experimental book version in everyday life. They may have experience with comic books and graphic novels though, in which they also have to integrate pictures and text to understand the storyline (Jacobs, 2007a,b). However, these stories are told by a sequence of images on each page, accompanied by captions in relatively small boxes or balloons. Our experimental book version, in contrast, contained full-page text blocks, alternated by one- or two-page picture spreads without any words. It would be interesting to examine whether imagery skills also play a role in understanding comic books and graphic novels.

## Conclusion

As texts are currently no longer the main source of information in everyday life and information becomes more and more multimodal, the need for adequate visual literacy and mental imagery skills becomes stronger and stronger (Walsh, 2003; Serafini, 2011; Ploetzner et al., 2013). Our findings indicate that interpreting pictures adequately is an ability that not all children possess. In addition, children differ in mental imagery skills, which are used to integrate words and pictures into one coherent mental model of the story. Future research should examine ways to effectively teach children how to interpret pictures that accompany or replace words and how to incorporate that information into their mental model of the story. Presumably, teaching children effective visual literacy and mental imagery strategies will lead to improved mental models and therefore enhanced story comprehension.

## Author Contributions

All authors (IB, SM, and JJ) confirm that they contributed to this paper by: Contributing substantially to the conception of the work (IB and SM); or the acquisition, analysis, and interpretation of the data (IB, SM, and JJ); drafting the work (IB) or revising it critically for important intellectual content (IB, SM, and JJ); approving the final version of the paper to be published (IB, SM, and JJ); agreeing to be accountable for all aspects of the work in ensuring that questions related to the accuracy or integrity of any part of the work are appropriately investigated and resolved (IB, SM, and JJ).

## Conflict of Interest Statement

The authors declare that the research was conducted in the absence of any commercial or financial relationships that could be construed as a potential conflict of interest.
