# Supplementary material for: Reading Pictures for Story Comprehension Requires Mental Imagery Skills
Source: Front Psychol. 2016 Oct 24;7:1630. doi: 10.3389/fpsyg.2016.01630 (PMC5075566; doi:10.3389/fpsyg.2016.01630)
Supplement: Supplementary file 1 [file Data_Sheet_1.pdf]

Appendix A Sample Pages of the Experimental Picture Version

Kodo opende zijn ogen. Hij lag op zijn vaders slaapmat. Met schaamte herinnerde hij zich de afstraffing. Vreemd genoeg deed zijn lijf geen pijn.
'Hoe is het met je, Onwetende?' Vanaf het papier van de fusama keek de Oude hem niet onvriendelijk aan.
Kodo ging rechtop zitten. Zijn armen en bovenlichaam waren in witte, linnen windsels gewikkeld. Er kwam een scherpe stank vanaf. Het vosje zat in een hoek.
'Bonenwrongel,' zei de Oude. 'Een wondermiddel als je een flink pak rammel hebt gekregen.'
'Het stinkt!' zei Kodo, die voorzichtig aan de windsels rook. Hij moest er bijna van kokhalzen.

Het vosje kwam naar hem toe en geeuwde. Kodo keek naar de kleine, scherpe tanden.
Ik moet sterker worden, dacht hij. Ik moet er klaar voor zijn als de Ninja's komen. Als ik elke dag oefen, word ik elke dag sterker. Hij stond op en stompte zichzelf op zijn voorhoofd. 'Ik word samoerai!'
'Laat mij je een welgemeende raad geven, Onwetende,' zei de Oude. 'Het gaat bij het zwaardvechten vooral om de ogen.'
'Bemoei je met je eigen zaken!' riep Kodo. 'Penseelkop!' slikte hij nog net in. Hij liep naar de fusama, schoof die open en keek om naar het vosje.
'Kom, Kitsune-chan, we gaan.'
'Ogen, Onwetende. Ogen!' riep de Oude hem na. 'Vergeet het niet. En kijk uit naar de kraanvogels.'

Toen Kodo buiten kwam, stond zijn vader aan de zijkant van het Veerhuis, bij de Rozenstruik. Hij droeg het Langzwaard op zijn rug, het Kortzwaard stak uit zijn gordel.
Kodo liep naar hem toe en keek hem aan. 'We moeten oefenen, Sensei*.' Hij boog zijn hoofd.
'Weet je zeker dat je het wilt?'
Kodo knikte.
Zijn vader verwijderde de windsels. Daarna waste hij Kodo's armen, zijn bovenlichaam en gezicht met sneeuw en knoopte hem een witte hoofdband om.
'Je kleren liggen in je kamer. Ik wacht hier op je.'

* Sensei: Meester

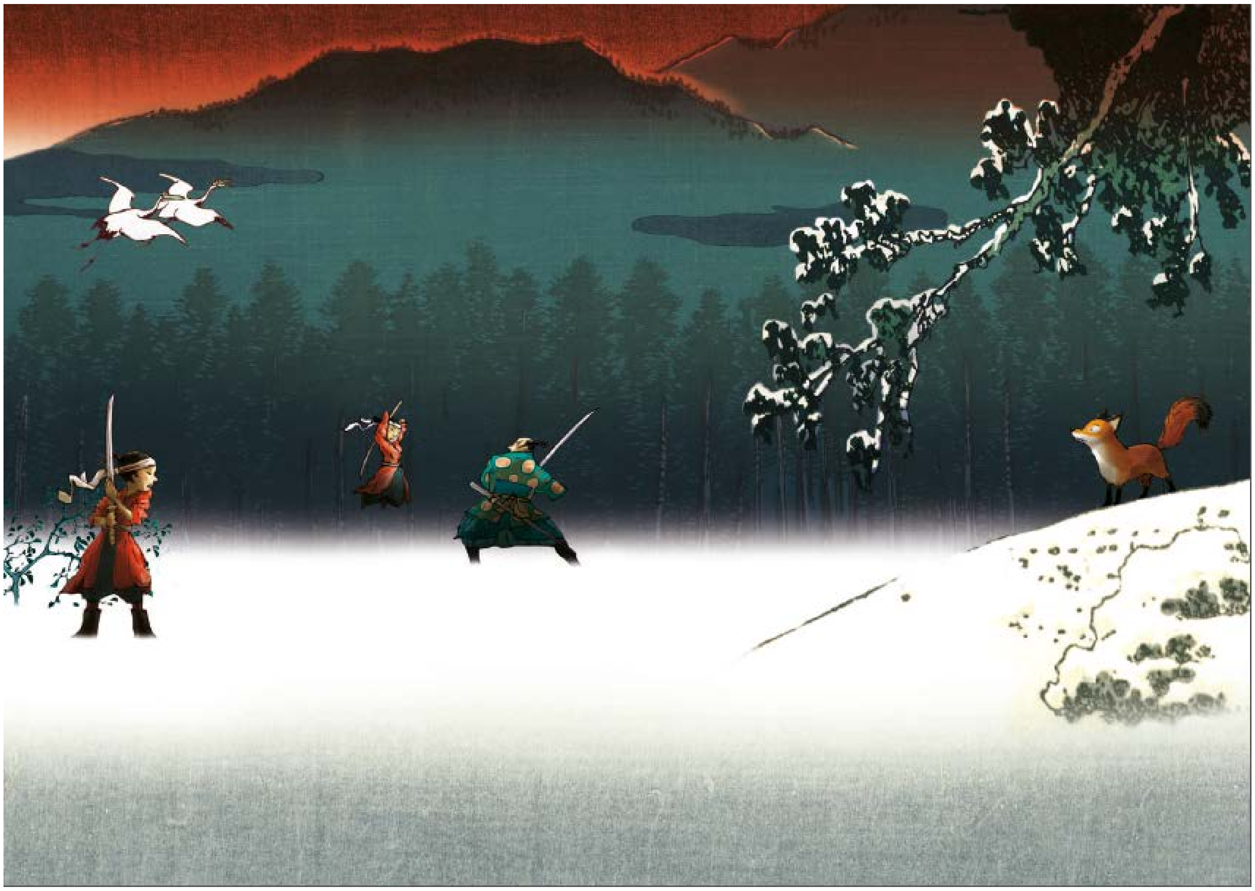

Kodo's vader keek omhoog. Hij liet zijn zwaard zakken en wees naar de lucht.
Daar naderden, in het rode licht van de ondergaande zon, twee grote witte vogels.
Ze landden vlakbij in de sneeuw.
'Kijk hoe ze dansen, Musko. De kraanvogels nemen afscheid van de Winter en begroeten de Lente.'
De vogels draaiden en hupten. Ze spreidden hun sterke vleugels en maakten lange passen en hoge sprongen. Ze bogen en strekten hun trotse, sierlijke halzen en wreven ze tegen elkaar.
'Kraanvogels kennen het geheim van geluk,' zei zijn vader. 'Elk paar blijft hun leven lang samen.'
Kodo dacht aan zijn moeder. 'Hoe moet het dan als een van hen er niet meer is?' vroeg hij.
Hij voelde de arm van zijn vader om zijn schouder. De zon ging onder.
Kodo huiverde. Vanaf de Overkant kwam hen, over het bevroren water, de zoete geur tegemoet die hij de vorige avond in de woonkamer had geroken.

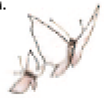

© 2014 Clavis Publishing, Hasselt (Belgium) – Amsterdam (the Netherlands), illustrations by Mark Janssen in Kodo. De weg van de Boog [Kodo. The road of the bow] written by Bert Kouwenberg

English translation:
Kodo opened his eyes. He was lying on his father's sleeping mat. He felt ashamed as he remembered the beatings. Strangely enough his body did not hurt. "How are you, Ignorant One?" From the paper of the fusama the Old Man looked up at him in a not unfriendly way. Kodo sat up straight. His arms and upper body were wrapped in white linen bindings. They created a sharp bad smell. The little fox was sitting in a corner. "Bean curd," the Old Man said. "A miracle cure when you have been beaten up." "It stinks!" Kodo said, who smelled the bindings cautiously. It almost made him gag. The little fox came over to him and yawned. Kodo looked at the small, sharp teeth. I need to get stronger, he thought. I need to be ready when the Ninjas come. If I practice every day, I will get stronger every day. He got up and slapped himself on the forehead. "I'm going to be a samurai!" "Let me give you some sincere advice, Ignorant One," the Old Man said. "In sword fighting it's all about the eyes." "Mind your own business!" Kodo yelled. He could just refrain himself from adding "Pencil head!". He walked towards the fusama, opened it and looked over his shoulder at the little fox. "Come, Kitsune-chan, let's go." "Eyes, Ignorant One. Eyes!" the Old Man shouted at him. "Don't forget that. And look for the cranes."

When Kodo came outside his father was standing next to the Ferry House, close to the Rose Bush. He carried the Long Sword on his back, the Short Sword was sticking out of his belt. Kodo walked towards him and looked at him. "We need to practice, Sensei*." He bowed his head. "Are you sure you want this?" Kodo nodded. His father removed the bindings. Then he washed Kodo's arms, his upper body and his face with snow and put a headband around his head. "Your clothes are in your room. I'll wait here for you."

Kodo's father looked up. He dropped his sword and pointed up to the sky. There, in the light of the setting sun, two white birds were approaching. They landed nearby in the snow. "Look at them dancing, Musko. The cranes are saying goodbye to the Winter and welcoming the Spring." The birds turned and hopped. They spread their strong wings, took big steps and made big jumps. They bent and stretched their proud and elegant necks and rubbed them against each other. "Cranes know the secret of happiness," his father said. "Each pair stays together for the rest of their lives." Kodo thought about his mother. "But what if one of them is gone?" he asked. He felt his father's arm over his shoulder. The sun was setting. Kodo shivered. From the Other

Side, over the frozen water, the sweet odor that he had smelled last night in the living room was coming closer.

*Sensei: master

Appendix B Sample Pages of the Pictures-and-Text Version

Kodo opende zijn ogen. Hij lag op zijn vaders slaapmat. Met schaamte herinnerde hij zich de afstraffing. Vreemd genoeg deed zijn lijf geen pijn.
'Hoe is het met je, Onwetende?' Vanaf het papier van de fusama keek de Oude hem niet onvriendelijk aan.
Kodo ging rechtop zitten. Zijn armen en bovenlichaam waren in witte, linnen windsels gewikkeld. Er kwam een scherpe stank vanaf. Het vosje zat in een hoek.
'Bonenwrongel,' zei de Oude. 'Een wondermiddel als je een flink pak rammel hebt gekregen.'
'Het stinkt!' zei Kodo, die voorzichtig aan de windsels rook. Hij moest er bijna van kokhalzen.
Het vosje kwam naar hem toe en geeuwde. Kodo keek naar de kleine, scherpe tanden.
Ik moet sterker worden, dacht hij. Ik moet er klaar voor zijn als de Ninja's komen. Als ik elke dag oefen, word ik elke dag sterker. Hij stond op en stompte zichzelf op zijn voorhoofd. 'Ik word samoerai!'
'Laat mij je een welgemeende raad geven, Onwetende,' zei de Oude. 'Het gaat bij het zwaardvechten vooral om de ogen.'
'Bemoei je met je eigen zaken!' riep Kodo. 'Penseelkop!' slikte hij nog net in. Hij liep naar de fusama, schoof die open en keek om naar het vosje. 'Kom, Kitsune-chan, we gaan.'

'Ogen, Onwetende. Ogen!' riep de Oude hem na. 'Vergeet het niet. En kijk uit naar de kraanvogels.'

Toen Kodo buiten kwam, stond zijn vader aan de zijkant van het Veerhuis, bij de Rozenstruik. Hij droeg het Langzwaard op zijn rug, het Kortzwaard stak uit zijn gordel.
Kodo liep naar hem toe en keek hem aan. 'We moeten oefenen, Sensei*.' Hij boog zijn hoofd.
'Weet je zeker dat je het wilt?'
Kodo knikte.
Zijn vader verwijderde de windsels. Daarna waste hij Kodo's armen, zijn bovenlichaam en gezicht met sneeuw en knoopte hem een witte hoofdband om.
'Je kleren liggen in je kamer. Ik wacht hier op je.'

---
* Sensei: Meester

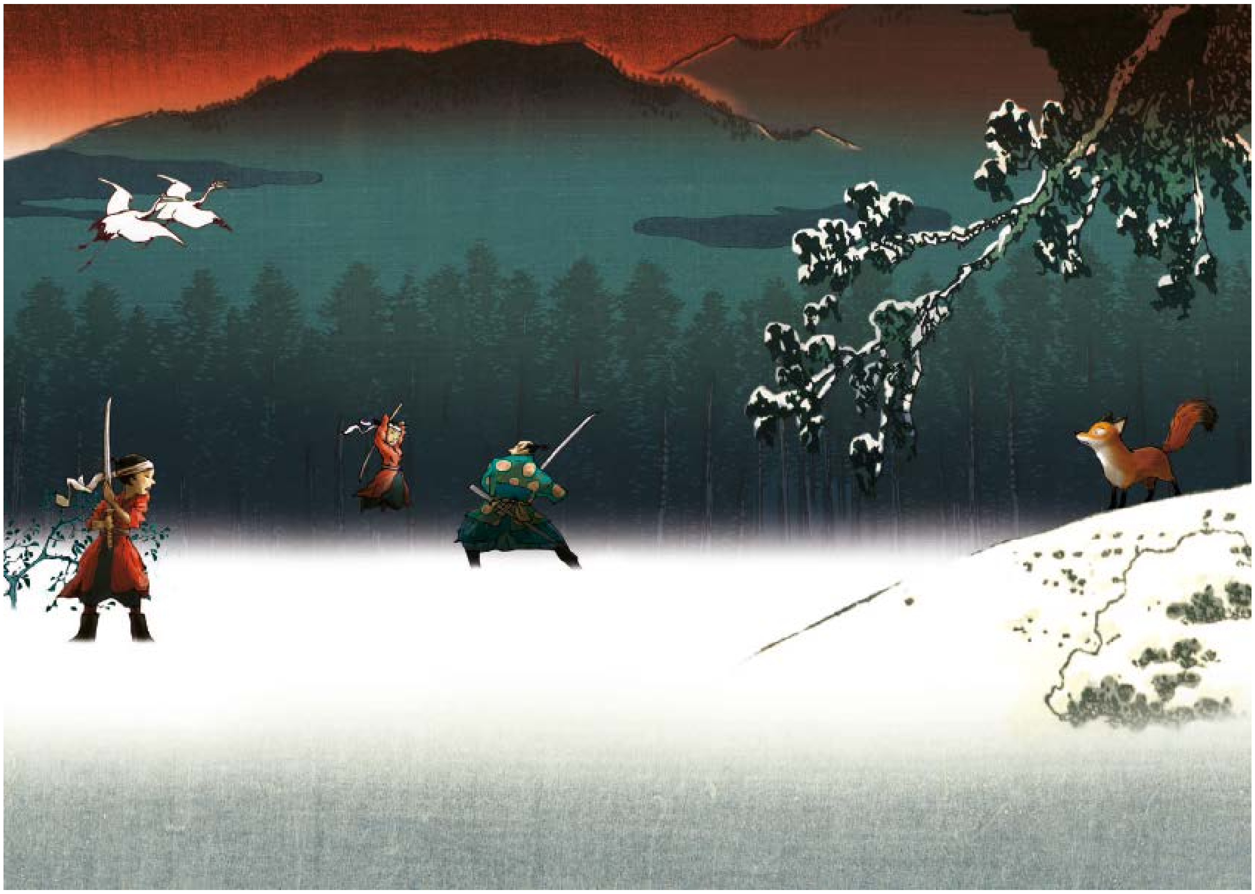

Met opgeheven zwaarden stonden ze tegenover elkaar. 'Ogen,' beval zijn vader. Kodo sperde zijn ogen wijd open, maar kon de blik van zijn vader niet lang weerstaan. Hij sloeg zijn ogen neer, liet zijn zwaard zakken en streek met zijn hand langs zijn wang.
'Opnieuw! Kijk in mijn ogen.'
'Het gaat mij lukken,' gromde Kodo.
Opnieuw hief hij het zwaard en keek zijn vader strak aan. Hij bleef staren tot hij er duizelig van werd. Zijn knieën knikten, zijn voeten leken weg te zinken in de sneeuw, het zwaard trok aan zijn armen.
'Ogen! Ogen!'
Kodo rechtte zijn rug. Een warme gloed trok door zijn hele lijf tot in het zwaard.
'Val aan.' riep zijn vader. 'Nu!'
Kodo sprong naar voren en haalde uit. Maar hij sloeg in de lucht, omdat zijn vader de aanval ontweek.
Zijn vader zei niets, maar knikte goedkeurend. Kodo wist dat hij geen groter compliment kon krijgen. De Oude van de fusama had gelijk gehad dat het bij zwaardvechten om de ogen gaat.

Kodo's vader keek omhoog. Hij liet zijn zwaard zakken en wees naar de lucht. Daar naderden, in het rode licht van de ondergaande zon, twee grote witte vogels. Ze landden vlakbij in de sneeuw.

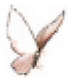

'Kijk hoe ze dansen, Musko. De kraanvogels nemen afscheid van de Winter en begroeten de Lente.'
De vogels draaiden en hupten. Ze spreidden hun sterke vleugels en maakten lange passen en hoge sprongen. Ze bogen en strekten hun trotse, sierlijke halzen en wreven ze tegen elkaar.
'Kraanvogels kennen het geheim van geluk,' zei zijn vader. 'Elk paar blijft hun leven lang samen.'
Kodo dacht aan zijn moeder. 'Hoe moet het dan als een van hen er niet meer is?' vroeg hij.
Hij voelde de arm van zijn vader om zijn schouder. De zon ging onder.
Kodo huiverde. Vanaf de Overkant kwam hen, over het bevroren water, de zoete geur tegemoet die hij de vorige avond in de woonkamer had geroken.

© 2014 Clavis Publishing, Hasselt (Belgium) – Amsterdam (the Netherlands), illustrations by Mark Janssen in Kodo. De weg van de Boog [Kodo. The road of the bow] written by Bert Kouwenberg

English translation:
Kodo opened his eyes. He was lying on his father's sleeping mat. He felt ashamed as he remembered the beatings. Strangely enough his body did not hurt. "How are you, Ignorant One?" From the paper of the fusama the Old Man looked up at him in a not unfriendly way. Kodo sat up straight. His arms and upper body were wrapped in white linen bindings. They created a sharp bad smell. The little fox was sitting in a corner. "Bean curd," the Old Man said. "A miracle cure when you have been beaten up." "It stinks!" Kodo said, who smelled the bindings cautiously. It almost made him gag. The little fox came over to him and yawned. Kodo looked at the small, sharp teeth. I need to get stronger, he thought. I need to be ready when the Ninjas come. If I practice every day, I will get stronger every day. He got up and slapped himself on the forehead. "I'm going to be a samurai!" "Let me give you some sincere advice, Ignorant One," the Old Man said. "In sword fighting it's all about the eyes." "Mind your own business!" Kodo yelled. He could just refrain himself from adding "Pencil head!". He walked towards the fusama, opened it and looked over his shoulder at the little fox. "Come, Kitsune-chan, let's go." "Eyes, Ignorant One. Eyes!" the Old Man shouted at him. "Don't forget that. And look for the cranes."

When Kodo came outside his father was standing next to the Ferry House, close to the Rose Bush. He carried the Long Sword on his back, the Short Sword was sticking out of his belt. Kodo walked towards him and looked at him. "We need to practice, Sensei." He bowed his head. "Are you sure you want this?" Kodo nodded. His father removed the bindings. Then he washed Kodo's arms, his upper body and his face with snow and put a headband around his head. "Your clothes are in your room. I'll wait here for you."

With raised swords they stood opposite each other. "Eyes," his father commanded. Kodo opened his eyes widely, but he couldn't resist his father's gaze for long. He cast down his eyes, lowered his sword and touched his cheek lightly with his hand. "Again! Look into my eyes." "I will succeed," Kodo grunted. He raised his sword again and stared at his father. He kept staring until he became dizzy. His knees were trembling, his feet seemed to sink in the snow, the sword

pulled on his arms. “Eyes! Eyes!” Kodo straightened his back. A warm glow spread through his whole body finishing in the sword. “Attack,” his father yelled. “Now!” Kodo jumped forward and attacked. But he hit into the air, because his father dodged the attack. His father did not say anything, but nodded approvingly. Kodo knew that he could not get a bigger compliment than that. The Old Man from the fusama had been right that sword fighting is all about the eyes.

Kodo’s father looked up. He dropped his sword and pointed up to the sky. There, in the light of the setting sun, two white birds were approaching. They landed nearby in the snow. “Look at them dancing, Musko. The cranes are saying goodbye to the Winter and welcoming the Spring.” The birds turned and hopped. They spread their strong wings, took big steps and made big jumps. They bent and stretched their proud and elegant necks and rubbed them against each other. “Cranes know the secret of happiness,” his father said. “Each pair stays together for the rest of their lives.” Kodo thought about his mother. “But what if one of them is gone?” he asked. He felt his father’s arm over his shoulder. The sun was setting. Kodo shivered. From the Other Side, over the frozen water, the sweet odor that he had smelled last night in the living room was coming closer.

*Sensei: Master

Appendix C Sample Pages of the Text-Only Version

Kodo opende zijn ogen. Hij lag op zijn vaders slaapmat. Met schaamte herinnerde hij zich de afstraffing. Vreemd genoeg deed zijn lijf geen pijn.
'Hoe is het met je, Onwetende?' Vanaf het papier van de fusama keek de Oude hem niet onvriendelijk aan.
Kodo ging rechtop zitten. Zijn armen en bovenlichaam waren in witte, linnen windsels gewikkeld. Er kwam een scherpe stank vanaf. Het vosje zat in een hoek.
'Bonenwrongel,' zei de Oude. 'Een wondermiddel als je een flink pak rammel hebt gekregen.'
'Het stinkt!' zei Kodo, die voorzichtig aan de windsels rook. Hij moest er bijna van kokhalzen.
Het vosje kwam naar hem toe en geeuwde. Kodo keek naar de kleine, scherpe tanden.
Ik moet sterker worden, dacht hij. Ik moet er klaar voor zijn als de Ninja's komen. Als ik elke dag oefen, word ik elke dag sterker. Hij stond op en stompte zichzelf op zijn voorhoofd. 'Ik word samoerai!'
'Laat mij je een welgemeende raad geven, Onwetende,' zei de Oude. 'Het gaat bij het zwaardvechten vooral om de ogen.'
'Bemoei je met je eigen zaken!' riep Kodo. 'Penseelkop!' slikte hij nog net in. Hij liep naar de fusama, schoof die open en keek om naar het vosje. 'Kom, Kitsune-chan, we gaan.'
'Ogen, Onwetende. Ogen!' riep de Oude hem na. 'Vergeet het niet. En kijk uit naar de kraanvogels.'

Toen Kodo buiten kwam, stond zijn vader aan de zijkant van het Veerhuis, bij de Rozenstruik. Hij droeg het Langzwaard op zijn rug, het Kortzwaard stak uit zijn gordel.
Kodo liep naar hem toe en keek hem aan. 'We moeten oefenen, Sensei*'. Hij boog zijn hoofd.

'Weet je zeker dat je het wilt?'
Kodo knikte.
Zijn vader verwijderde de windsels. Daarna waste hij Kodo's armen, zijn bovenlichaam en gezicht met sneeuw en knoopte hem een witte hoofdband om.
'Je kleren liggen in je kamer. Ik wacht hier op je.'

Met opgeheven zwaarden stonden ze tegenover elkaar. 'Ogen,' beval zijn vader. Kodo sperde zijn ogen wijd open, maar kon de blik van zijn vader niet lang weerstaan. Hij sloeg zijn ogen neer, liet zijn zwaard zakken en streek met zijn hand langs zijn wang.
'Opnieuw! Kijk in mijn ogen.'
'Het gaat mij lukken,' gromde Kodo.
Opnieuw hief hij het zwaard en keek zijn vader strak aan. Hij bleef staren tot hij er duizelig van werd. Zijn knieën knikten, zijn voeten leken weg te zinken in de sneeuw, het zwaard trok aan zijn armen.
'Ogen! Ogen!'
Kodo rechtte zijn rug. Een warme gloed trok door zijn hele lijf tot in het zwaard.
'Val aan.' riep zijn vader. 'Nu!'
Kodo sprong naar voren en haalde uit. Maar hij sloeg in de lucht, omdat zijn vader de aanval ontweek.
Zijn vader zei niets, maar knikte goedkeurend. Kodo wist dat hij geen groter compliment kon krijgen. De Oude van de fusama had gelijk gehad dat het bij zwaardvechten om de ogen gaat.

Kodo's vader keek omhoog. Hij liet zijn zwaard zakken en wees naar de lucht. Daar naderden, in het rode licht van de ondergaande zon, twee grote witte vogels. Ze landden vlakbij in de sneeuw.
'Kijk hoe ze dansen, Musko. De kraanvogels nemen afscheid van de Winter en

* Sensei: Meester

begroeten de Lente.'
De vogels draaiden en hupten. Ze spreidden hun sterke vleugels en maakten lange passen en hoge sprongen. Ze bogen en strekten hun trotse, sierlijke halzen en wreven ze tegen elkaar.
'Kraanvogels kennen het geheim van geluk,' zei zijn vader. 'Elk paar blijft hun leven lang samen.'
Kodo dacht aan zijn moeder. 'Hoe moet het dan als een van hen er niet meer is?' vroeg hij.
Hij voelde de arm van zijn vader om zijn schouder. De zon ging onder.
Kodo huiverde. Vanaf de Overkant kwam hen, over het bevroren water, de zoete geur tegemoet die hij de vorige avond in de woonkamer had geroken.

© 2014 Clavis Publishing, Hasselt (Belgium) – Amsterdam (the Netherlands), illustrations by Mark Janssen in Kodo. De weg van de Boog [Kodo. The road of the bow] written by Bert Kouwenberg

English translation:
Kodo opened his eyes. He was lying on his father's sleeping mat. He felt ashamed as he remembered the beatings. Strangely enough his body did not hurt. "How are you, Ignorant One?" From the paper of the fusama the Old Man looked up at him in a not unfriendly way. Kodo sat up straight. His arms and upper body were wrapped in white linen bindings. They created a sharp bad smell. The little fox was sitting in a corner. "Bean curd," the Old Man said. "A miracle cure when you have been beaten up." "It stinks!" Kodo said, who smelled the bindings cautiously. It almost made him gag. The little fox came over to him and yawned. Kodo looked at the small, sharp teeth. I need to get stronger, he thought. I need to be ready when the Ninjas come. If I practice every day, I will get stronger every day. He got up and slapped himself on the forehead.

“I’m going to be a samurai!” “Let me give you some sincere advice, Ignorant One,” the Old Man said. “In sword fighting it’s all about the eyes.” “Mind your own business!” Kodo yelled. He could just refrain himself from adding “Pencil head!”. He walked towards the fusama, opened it and looked over his shoulder at the little fox. “Come, Kitsune-chan, let’s go.” “Eyes, Ignorant One. Eyes!” the Old Man shouted at him. “Don’t forget that. And look for the cranes.”

When Kodo came outside his father was standing next to the Ferry House, close to the Rose Bush. He carried the Long Sword on his back, the Short Sword was sticking out of his belt. Kodo walked towards him and looked at him. “We need to practice, Sensei.” He bowed his head. “Are you sure you want this?” Kodo nodded. His father removed the bindings. Then he washed Kodo’s arms, his upper body and his face with snow and put a headband around his head. “Your clothes are in your room. I’ll wait here for you.”

With raised swords they stood opposite each other. “Eyes,” his father commanded. Kodo opened his eyes widely, but he couldn’t resist his father’s gaze for long. He cast down his eyes, lowered his sword and touched his cheek lightly with his hand. “Again! Look into my eyes.” “I will succeed,” Kodo grunted. He raised his sword again and stared at his father. He kept staring until he became dizzy. His knees were trembling, his feet seemed to sink in the snow, the sword pulled on his arms. “Eyes! Eyes!” Kodo straightened his back. A warm glow spread through his whole body finishing in the sword. “Attack,” his father yelled. “Now!” Kodo jumped forward and attacked. But he hit into the air, because his father dodged the attack. His father did not say anything, but nodded approvingly. Kodo knew that he could not get a bigger compliment than that. The Old Man from the fusama had been right that sword fighting is all about the eyes.

Kodo’s father looked up. He dropped his sword and pointed up to the sky. There, in the light of the setting sun, two white birds were approaching. They landed nearby in the snow. “Look at them dancing, Musko. The cranes are saying goodbye to the Winter and welcoming the Spring.” The birds turned and hopped. They spread their strong wings, took big steps and made big jumps. They bent and stretched their proud and elegant necks and rubbed them against each other. “Cranes know the secret of happiness,” his father said. “Each pair stays together for the rest of their lives.” Kodo thought about his mother. “But what if one of them is gone?” he asked. He felt his father’s arm over his shoulder. The sun was setting. Kodo shivered. From the Other Side, over the frozen water, the sweet odor that he had smelled last night in the living room was coming closer.

*Sensei: Master

Appendix D Example Questions of the Story Comprehension Test

Example 1. Why does the first training end?

A. **Kodo is in so much pain that he faints.*
B. Kodo asks his father to stop the training.
C. Kodo's father wants to stop the training.
D. Kodo is able to beat his father one time.

Example 2. When Kodo wakes up after the first training his body is wrapped around with white linen bindings that smell really bad. Why?

A. **To make sure he is not in pain.*
B. To keep him sleeping as much as possible.
C. To tie him to his sleeping mat.
D. To make him gag.

Example 3. What is the old man's advice about sword fighting?

A. It's about speed.
B. **It's about the eyes.*
C. It's about the hands.
D. It's about the power.

Example 4. What secret do the cranes know?

A. The secret that Kodo's father is hiding from him.
B. The secret of the sweet smell.
C. **The secret of happiness.*
D. The secret of the four seasons.
